# Are virtual reality (VR) interventions for substance use disorders designed for implementation? A rapid review of platform characteristics and clinical fit

**DOI:** 10.21203/rs.3.rs-10501103/v1

**Published:** 2026-07-29

**Authors:** Chanda Phelan, Tyler Wray

**Affiliations:** Brown University; Brown University

**Keywords:** Virtual reality, Substance use disorder treatment, Implementation science, Clinical implementation barriers, Rapid review, Consolidated Framework for Implementation Research (CFIR)

## Abstract

**Background:**

Virtual reality (VR) interventions show promise for improving substance use disorder (SUD) treatment outcomes, but successful implementation depends on compatibility with real-world clinical settings. No review has yet characterized the implementation readiness of recently described SUD-focused VR platforms.

**Methods:**

We conducted a rapid review of peer-reviewed research published between 2020 and 2025 to characterize the implementation readiness of recently described SUD-focused VR platforms, focusing on their technical characteristics, therapeutic goals, and implementation requirements. Following a systematic search, 14 studies were included. Each intervention was coded for platform features such as hardware requirements, interactivity, and clinician involvement using a framework informed by the Consolidated Framework for Implementation Research (CFIR).

**Results:**

Patient acceptability and tolerability were consistently high across studies. However, several platform characteristics posed potential implementation barriers. Four studies (29%) required PC-tethered hardware or specialized physical spaces that could hinder adoption. A minority (n = 5, 36%) were explicitly grounded in established evidence-based approaches. Ten (71%) were designed primarily as self-guided supplemental interventions largely independent of routine treatment sessions.

**Conclusions:**

Most recently described VR interventions for SUD have implementation barriers that could limit adoption in community treatment settings. We recommend that developers design for portable, standalone hardware, keep session length and training burden realistic for community settings, ground therapeutic content in familiar evidence-based practices, and favor interactive over passive designs. Addressing these factors proactively will help ensure VR experiences for SUD treatment align with the practical realities of the settings in which they will be used.

## Introduction

Substance use disorders (SUD) are among the top causes of disability globally and have immense social and economic costs.^1,2^ Treatment is effective^3–5^, but 40–60% of those who receive treatment relapse within the first year.^6^ Finding ways to improve the efficacy of SUD treatment could help more people enter and sustain recovery^7,8^ and is an important research priority.

Incorporating virtual reality (VR) experiences into SUD counseling may be one way of improving outcomes in addiction treatment. VR refers to a computer-generated experience that provides an extensive, surrounding, and vivid illusion of reality to human users.^9^ VR is unique among technologies in that it gives users a convincing feeling of *presence*, or a subjective sense that they are actually *in* the digital world.^10^ It achieves this effect by providing users with sensory input that mimics the way humans naturally explore and navigate the world (e.g., stereoscopic vision, head movement/tracking, stereo sound). This capacity to take patients beyond the therapy room and creating a realistic sense of presence in a variety of therapeutically-relevant scenarios has led to its use in treatments for a variety of mental health conditions.^11^ However, the most mature application of VR to mental health treatment is in anxiety disorders. In these applications, counselors have used VR experiences to facilitate techniques that are foundational to most evidence-based, first-line treatments for anxiety: exposures.^12–14^ Exposure techniques involve gradually immersing patients in feared situations to help them confront fear-provoking stimuli to promote new safety learning that contradicts their anxious beliefs about their dangerousness and intolerability. Using VR to facilitate these exposures rather than the previously-common *in vivo* (i.e., real life) approach allows counselors greater flexibility and reduces many of the logistical barriers to delivering exposures (e.g., time, planning, travel).^15^

Strengths like these have increased interest in exploring applications of VR to addiction treatment. Many previous reviews have described a wide variety of ways VR has been used to augment or facilitate treatment, including helping patients identify triggers or practice coping strategies^16–18^, learn and practice mindfulness skills^17,19^, or to facilitate cue exposure^20^, counter-conditioning^21^, or cognitive bias modification.^22^ These reviews showed that incorporating VR into counseling-based SUD treatments is generally feasible but that, while preliminary evidence of efficacy for some uses of VR is promising, the rigor of efficacy studies has so far been limited by small sample sizes and few longitudinal studies of its impact on outcomes directly relevant to SUD, such as long-term abstinence and relapse rates.^17,23–25^ This research highlights the need for more rigorous evaluation of VR-assisted treatments for SUD.

However, just as important as efficacy is the question of implementation readiness: whether SUD-focused VR platforms have characteristics that would allow them to be feasibly and sustainably integrated into real-world clinical settings. As research on VR-assisted treatments for anxiety has shown, even platforms with demonstrated efficacy will struggle to gain traction among practicing counselors if they cannot be easily integrated into routine practice. The Consolidated Framework for Implementation Research (CFIR) is one of the most popular frameworks used in implementation science because its detailed accounting of constructs at various levels helps researchers identify factors that could affect implementation of a given innovation.^26^ CFIR suggests that there are several characteristics of the innovation itself that often determine whether it can be successfully implemented, including its complexity, adaptability, design, and cost. Several attributes that are unique to VR are likely to contribute to its overall implementation readiness, as well. For example, VR-assisted SUD treatment experiences that use hardware systems that require headsets to be physically tethered to a computer or require it to be used in a physical space that has mounted hardware, such as the HTC Vive Pro 1 or 2, would be considerably more complex to implement than those that use systems that are untethered and can be used in any space, such as the Oculus Quest ecosystem. The extent that counselors can customize and tailor each experience, and use them in sessions flexibly, would also increase their adaptability and likely facilitate implementation.

Considering the implementation context while designing innovations could also help ease barriers present at other levels of CFIR domains as well, particularly aspects of the “inner setting,” or characteristics of the implementing organization.^26^ For example, VR-assisted SUD experiences that are intended to complement treatment techniques that counselors already know and are familiar with would likely decrease the amount of knowledge and information that are needed for counselors to be able to successfully use it. Many other organizational factors play an important role, as well, including workflow fit and time burden^27,28^. These key considerations are echoed in previous reviews of VR implementation in other areas of healthcare, which have shown that many existing VR interventions often have salient implementation barriers within both the inner and outer setting levels, such as inadequate technical support, high equipment costs, unclear reimbursement pathways, and a lack of clinician confidence.^27,29^ Evidence from other clinical contexts also highlights the importance of targeted clinical training and explicit treatment protocols for using VR.^30,31^ Together, this work underscores that, if VR treatments for SUD are to add clinical value in the real world, they must be explicitly designed with the implementation context in mind and address key barriers that are likely to impede adoption and use.

In this rapid review, we synthesized research describing SUD-focused VR experiences published over the last five years to explore the potential compatibility of recently-described platforms with the contexts they are likely to be implemented in: community SUD treatment facilities. To our knowledge, no review to date has specifically analyzed the various characteristics of existing SUD VR experiences to assess implementation readiness and explore factors that could be barriers to implementation in this way. We elected to focus primarily on “platform characteristics,” meaning characteristics of the innovations themselves, since these are most thoroughly described in many publications on SUD-focused VRs and are potentially addressable through design. However, we also consider how these and other characteristics of the intended use scenario could impact implementation, including the need for (and extensiveness of) counselor and patient training, acceptability, and therapeutic goals.

## Methods

**Figure 1:**
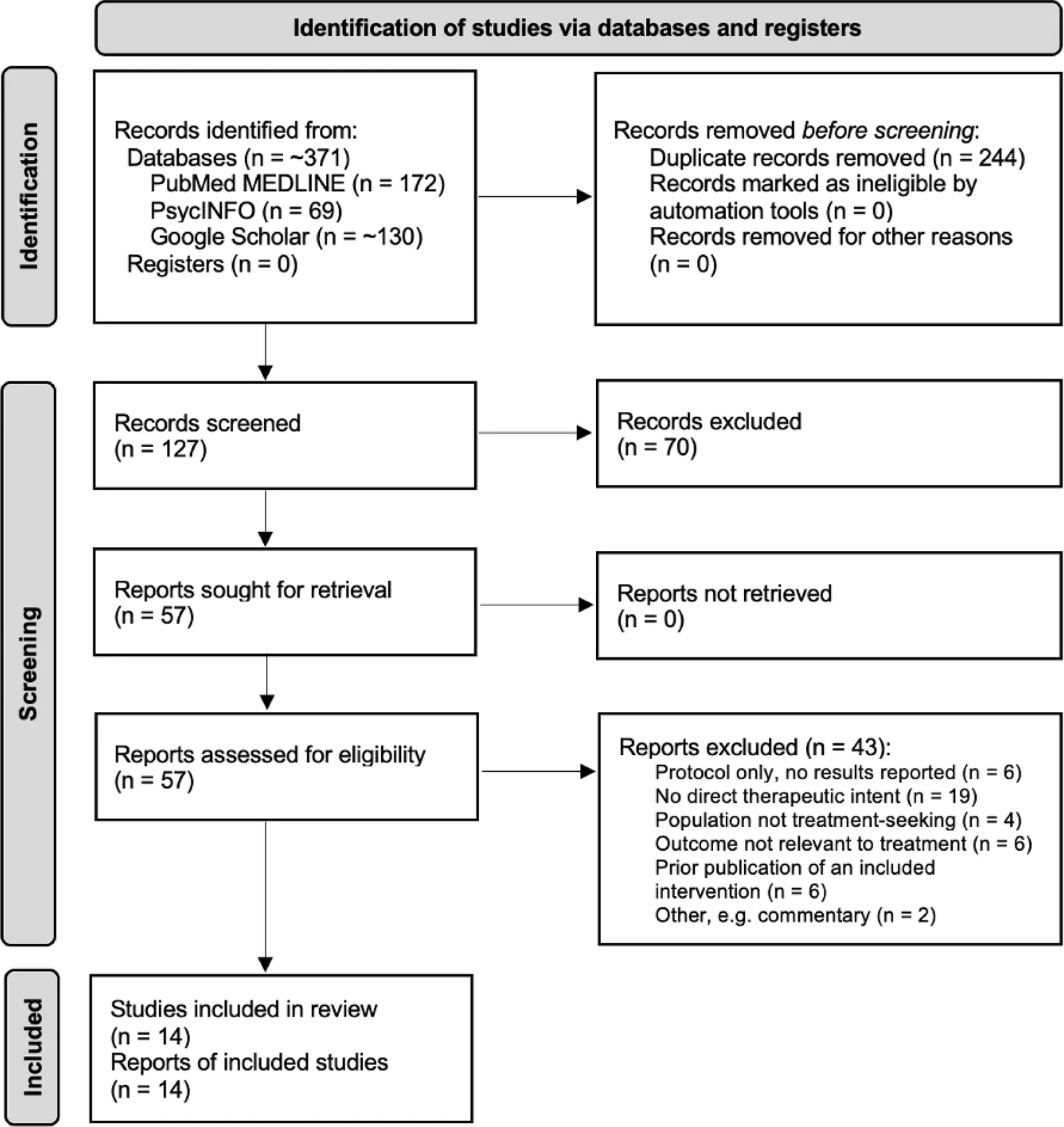
PRISMA flow diagram of study selection.

To identify relevant literature, we searched PubMed MEDLINE, PsycINFO and Google Scholar for articles published between January 2020 and October 2025, choosing a 5-year time frame to provide a snapshot of the most recent platforms and technological developments. We using the following search: (“virtual reality” OR VR) AND (“substance use disorder” OR “drug use” OR “alcohol use disorder” OR “opioid use disorder” OR “AUD” OR “OUD” OR “SUD” OR “addiction treatment” OR “substance abuse treatment”) AND (treatment OR intervention OR therapy OR rehabilitation OR “behavioral therapy” OR “relapse prevention” OR “craving reduction” OR “treatment retention” OR “use reduction”) NOT (smoking OR nicotine OR tobacco OR cigarette OR vape OR vaping OR “behavioral addiction” OR gambling). Additional records were identified through manual screening of reference lists from recent reviews. Titles and abstracts of all identified records were screened against inclusion and exclusion criteria, and full texts were retrieved for all records that passed this initial screen. One author conducted all screening, with the second author independently reviewing inclusion decisions. Disagreements were resolved through discussion. The full screening process is documented in the PRISMA flow diagram (Figure 1).^32^ Ethics approval was not required for this study, as it is a rapid review of previously published literature and did not involve the collection of new data from human participants. This review and protocol was not registered.

### Inclusion & Exclusion

#### Inclusion criteria.

Studies were included if the following criteria were met: 1) Direct therapeutic intent: The VR intervention was explicitly designed to modify an SUD-related outcome (e.g., abstinence, reduction in use, or improved treatment retention). 2) Clinical population: The intervention was developed for and tested in a population currently engaged in or seeking treatment for SUD. In instances where multiple publications reported on the same intervention (e.g., an initial feasibility pilot followed by a later randomized controlled trial), we retained only the most recent and methodologically descriptive publication.

#### Exclusion criteria.

Studies were excluded if they 1) focused exclusively on tobacco or nicotine use or behavioral addictions (e.g., gambling), 2) utilized VR solely for cue-reactivity assessment without a therapeutic component (i.e., for research), or 3) focused on general cognitive rehabilitation, such as executive function training, without an explicit goal of changing addiction-focused outcomes. We excluded smoking or nicotine-focused VR experiences since treatment for these conditions now rarely happens within structured treatment facilities.

### Coding approach

Studies that fit inclusion criteria were coded on a number of dimensions, many of which approximately correspond to CFIR constructs (see Table 1). To ensure rigor of the coding process, we employed researcher triangulation.^33^ The two authors coded the studies, with one completing initial coding of all papers, which was then independently reviewed by the author with clinical expertise in substance use disorders, who verified the accuracy of all codes and flagged any disagreements. The authors then met to resolve discrepancies and arrive at the final codes.

#### Study characteristics.

Various characteristics of each study were abstracted, such as the reported study’s basic design, the condition of focus, geographic location, treatment setting, sample size and composition, and follow-up period length.

#### VR platform and system characteristics.

Various characteristics of the VR experiences were coded, including the hardware ecosystem they used (e.g., HTC Vive, Oculus Quest, etc.) and whether the proposed experience would require any other infrastructure other than the headsets themselves, such as a tethered computer, mounted base stations, or separate observation rooms. We also coded the recommended intervention “dosage,” such as the number and length of VR sessions; what type of in-session navigation they used (e.g., stationary sitting, versus free movement); and whether VR experiences appeared to involve “active” interactions, meaning that users could interact with elements in the digital world in some way (e.g., move around objects or people, pick up and inspect objects, have a dialog with an avatar, etc.), versus those that involved mostly passive interaction (e.g., watching a 360 video). Finally, we also coded whether VR experiences appeared to depict or involve one or more common elements, such as drug-related cues, social situations, or nature scenes. The categories for this code were created as we read the full text of analyzed articles, with new categories created for experiences that involved elements that were not yet well-represented by previous ones.

#### Therapeutic goals.

We coded the underlying theoretical models that inspired each experience, if it was explicitly referenced or discussed in each publication. Guided by the National Institute on Drug Abuse’s *Principles of Addiction Treatment*^34^ and McGovern and Carroll’s^35^ review of evidence-based practices for substance use disorders, we also coded whether or not the stated therapeutic purposes of each VR experience was rooted in an approach to treatment that has been the focus of rigorous evaluation research and has received relatively strong empirical support. Experiences were coded as “yes” if the manuscripts describing them clearly linked them with behavior change techniques that are commonly used in at least one of the treatment approaches identified in these previous reviews.

#### Clinician roles and training.

For each analyzed study, we coded whether a given experience was likely to be *therapist guided* or not, with this variable coded “yes” if articles explicitly noted that therapists were actively involved in manipulating elements of the experience, discussing its content with patients, or were otherwise essential to the therapeutic impact of the experiences. They were coded “no” if articles did not note a specific, active role for therapists *during* or *immediately after* the experience or if it was clearly intended to be self-guided by patients. We also extracted any information about how much and what kind of training counselors needed to deliver the experience, if noted.

#### Patient training, acceptability, and usability.

We extracted information related to how much and what kind of training that patients might need in order to use each experience, if any. We also extracted any data included in any study about whether or not each experience was acceptable to patients (e.g., satisfaction), whether they found it to be usable, and whether any experienced adverse events.

#### Implementation context.

Finally, we coded a several organizational factors that could play a role in adoption, including how each experience was intended to fit into clinical workflows: Whether it was intended to be used by patients on a self-guided basis, under the active supervision of a counselor in-session, or outside of session with supervision by other clinical staff. We also coded whether each experience was intended to be used as an adjunct to standard treatment, a stand-alone tool, or as a fully embedded component within a specific approach to treatment. Lastly, we extracted barriers to implementation that each study reported that were unique to VR interventions, if any. Table 1 summarizes the coded characteristics and the CFIR domains and constructs to which they are mapped.

## Results

The PRISMA flow diagram (Figure 1) summarizes the search and screening process. Database searching yielded 127 records after deduplication. Based on title and abstract review, 104 were excluded for not meeting eligibility criteria. We reviewed the full text of 23 articles, and ultimately excluded nine additional studies for failing to meet inclusion criteria, including three studies that focused on general executive function training, rather than changing outcomes related to substance use. Fourteen studies were included in our final analysis.

Of the 14 studies in our final analysis (see Table 2), six were randomized controlled trials, five were single-arm studies with some degree of prospective follow-up, and three were single-session evaluations. Four focused on participants with alcohol use disorder, four on opioid use disorder, three included any kind of substance use disorder, two on stimulant (methamphetamine) use disorder, and one focused on cannabis use disorder among participants with severe mental illness. Eight were conducted in inpatient or residential treatment facilities, five were conducted in outpatient settings, and two were conducted in both inpatient/residential or outpatient settings. Seven studies took place in the United States, three in the European Union, two in China and one in Canada. Sample sizes ranged from 9 to 89 (*M*=39.9, *SD*=25.9), and among the five studies that had a defined follow-up period, those periods ranged from 1 month to 1 year (*M*=1.9 months, *SD*=4.3).

### Platform and system characteristics

#### Hardware.

Six of the interventions used standalone, mobile VR hardware. Three of these used Meta/Oculus headsets (Oculus Go, Oculus Quest, Meta Quest 2.0), one used PICO G2, one used specialized clinical hardware (REAL systems i-series), and two did not specify. Four of the interventions used PC-tethered VR hardware. Of these, two used Meta/Oculus headsets (Oculus Rift S, Meta Quest 3 in tethered mode), one used an unspecified Windows Mixed Reality headset, and one used Samsung Odyssey. The remaining four interventions did not specify whether their hardware was standalone or tethered. Table 3 summarizes system characteristics, including hardware.

#### Recommended “dosage.”

The dosage and duration of VR interventions varied considerably. Five were intended to be used in a single session, which was typically used for 15 minutes or less. An additional five were intended to be used from 8 to 24 times over periods of 4–12 weeks, with individual session lengths between 45 and 90 minutes. Three studies utilized a “burst” approach involving daily sessions over 1 to 3 weeks. One study employed a self-paced model, allowing patients to dictate their own dosage throughout their residential stay. Overall, among the 11–12 studies that reported sufficient information, the total minutes of exposure ranged from six to 600 minutes (*M*= 251.0, *SD*=241.9) over a period of one to 84 days (*M*=29.2, *SD*=26.6).

#### Interactivity.

The degree of interactivity was split evenly between the interventions, with seven involving active elements, and the other seven interventions either utilizing a passive (n=6) or semi-active (n=1) approach to interaction. One passive intervention used a rendered 3D environment,^36^ while the remainder (n=5) used 360 video, consisting of pre-shot scenes or pre-recorded guided meditations where the user had limited control beyond selecting which module to experience.^37–41^ The sole semi-active intervention used a rendered 3D environment, but with more interactivity: participants primarily experienced scripted avatar monologues with gaze-triggered scene transitions and a single narrative choice point between two possible futures.^42^

Active interaction designs typically required users to influence the virtual environment through physical movements, such as choreographed martial-arts motor sequences in Faraj et al.^43^; or via complex narrative participation, such as the real-time dialogues with live therapist-controlled avatars in Giguère et al.^44^ In other active interventions, patients manipulated objects in the virtual environment, such as handling beverages or navigating a virtual supermarket to practice using coping skills.^45–47^ Active interaction design was most frequently used for practicing skills (n=4) or immersive cue exposure (n=3). Passive interaction design was most frequently used for mindfulness/stabilization (n=4) or classic cue exposure (n=2), where the goal was immersive observation rather than environmental manipulation.

#### In-experience navigation.

Thirteen of the 14 experiences (92%) were designed to be used in a stationary sitting position. Only Landhäußer et al.^47^ used physical locomotion with a free-walking system where the patients’ actual steps in a 5m × 5m physical space were mapped into the virtual environment. Only one other intervention (Wiese et al.^41^) incorporated virtual movement, allowing patients to “walk” through a virtual wooded retreat using a hand controller. Faraj et al.^43^ used active body engagement through a choreographed martial-arts sequence, but only in the upper body; patients’ feet never moved. Of the remaining 11 interventions, three used handheld controllers to manipulate objects in the virtual world. The remaining interventions were completely stationary and involved no user-controlled movement within the experience. To enhance immersion without requiring movement, Shen et al.^42^ used a fully rendered 3D environment with a physical park bench co-registered in both digital and physical space. Participants briefly stood in front of a virtual mirror to establish body transfer before being seated, and navigated between scenes via gaze-triggered teleportation.

#### Experience categories & descriptions.

All 14 included studies’ VR experiences incorporated the same five broad categories of content, which included: nature scenes (n=5), social situations such as a restaurant or bar (n=7), and drug cues (n=8). Less common shared elements were mindfulness support (n=3) or experiencing negative consequences (n=2).

#### Additional infrastructure needs.

Four included studies (28.6%) described experiences that required additional equipment or infrastructure beyond the computers needed for tethering that some experiences required (described in the “Hardware” subsection above). One required a private soundproof room^43^, one required two adjacent rooms separated by a one-way mirror so the clinician could monitor the patient^44^, one required a “free-walking” interaction method that required a dedicated 5m × 5m navigation space^47^, and one required a steel park bench, to match the tactile experience with the virtual environment.^42^ For specialized peripherals and other tools, five studies (35.7%) integrated wearable biofeedback hardware, including the Empatica E4 wristband^47^, smartwatches^48^, or electronic sphygmomanometers to collect objective physiological data like heart rate.^40^ Three interventions utilized olfactory stimulation tools: two used real alcohol with cotton pads^39,45^, and one used a scent diffuser delivering the smell of newly mowed grass.^42^

### Therapeutic goals

#### Theoretical models used.

Most interventions drew from one of three primary theoretical frameworks: cue exposure and extinction-based approaches (n=6), cognitive behavioral approaches (n=6), and mindfulness-based approaches (n=5). Cue exposure interventions typically leveraged classical conditioning and extinction learning mechanisms to reduce craving through repeated exposure to substance-related cues. Experiences drawing from principles of cognitive behavioral therapy (CBT) generally helped patients identify maladaptive beliefs related to high-risk situations and practice using coping skills. Mindfulness experiences encouraged patients to practice mindfulness skills, like forgiveness, gratitude, body scanning, and breathing exercise. Five interventions integrated multiple frameworks, such as Mindfulness-Oriented Recovery Enhancement^48^, which combined mindfulness training, cognitive reappraisal, and cue exposure mechanisms, and 3D Therapy Thrive^46^, which integrated mindfulness-based cognitive therapy with elements of CBT. The two interventions using other theoretical frameworks included one that targeted future self-continuity and delay discounting through intertemporal choice theory^42^, and another that used martial arts-based therapy to “modulate the pain neuromatrix.”^43(p. 2739)^

#### Alignment with established evidence-based practices.

Five of the publications we reviewed noted that the VR interventions they developed were explicitly or directly inspired by or intended to complement techniques or approaches to treatment that are widely recognized to be evidence-based.^34,35^ Publications describing three additional interventions noted some inspiration from techniques considered evidence-based, but also new or additional approaches. For example, Weise et al. (2024) reported that their experience was based on a new approach they called Transcending Self Therapy, which itself drew inspiration from Cognitive Behavioral Therapy (CBT). Of the remaining six interventions that were classified as having limited alignment with evidence-based practices, five were classified as such because they were based on cue exposure therapy (CET). Table 4 summarizes therapeutic models and connection to established evidence-based practices.

### Clinician roles and training

#### Clinician role.

Most interventions (n=10) did not specify an active role for clinicians during or immediately after the VR experience, with the majority of these interventions instead describing an experience that was patient-guided or system-guided in a treatment setting where clinicians were present only for safety monitoring. Only four interventions were clinician-guided. In Thaysen-Petersen et al.^49^, nurses explicitly performed cognitive analysis during or immediately after patients interacted with the VR experience. Ji et al.^40^ employed a similar strategy. In Giguère et al.^44^, the clinician is even more involved, piloting the movements, facial expressions and speech of an avatar “reflecting a significant person associated with [participants’] cannabis use” that interacts with the patient during the VR experience. Lastly, the martial arts-based mindfulness intervention Faraj et al.^43^ had a clinician do one-on-one training and facilitation with the patient during the VR session.

#### Clinician training needs.

The four clinician-guided interventions required extensive training, with Thaysen-Petersen et al.^49^ representing the highest training burden, with 49 hours of training for the manualized treatment. Ji et al.^40^ also required specialized training that included modeling intervention scenarios and preparing for potential complications, though the time required for the training was unspecified. Giguère et al.^44^ and Faraj et al.^43^ required specialized skills: acting and martial arts movement, respectively. Though the remaining interventions were largely self-guided, many of them still required the clinician to manage a specific therapeutic sequence, orient the patient, and/or handle any potential distress.^37,39,45,48^ Others required brief (less than 30 minute) orientation on the therapeutic sequence and/or how to teach the patient to use the VR equipment.^36,38,41,46^ Only one required no training beyond a technical orientation, which used an off-the-shelf commercial application (Calm) that minimized training needs.^36^

### Patient training, acceptability, and usability

#### Patient training needs.

The reviewed interventions had several tiers of patient training needs. For half of the interventions (n=7), patients needed only a brief orientation to teach controls and introduce the intervention, with one of these requiring slightly more training because the interaction methods were more complex and included free-walking.^47^ Two interventions required significant preparatory work up front to personalize their avatars, provide voice samples and supply other data to personalize the intervention experience.^42,44^ The remaining five interventions required some level of conceptual training on top of technical training before embarking on the VR experience. Faraj et al.^43^, for example, needed patients to memorize a specific mantra and set of physical movements before the VR experience. Few publications explicitly reported the specific amount of time that was required for patient training.

#### Patient acceptability.

Thirteen of the 14 studies assessed patient acceptability or satisfaction, though measurement approaches varied considerably. Seven studies utilized quantitative satisfaction or acceptability scales, including validated instruments such as the Net Promoter Score^48^ and the Theoretical Framework of Acceptability.^38^ Eight studies gathered qualitative feedback through semi-structured interviews or open-ended assessments. Several studies combined both quantitative scales and qualitative interviews to evaluate the patient experience. Overall, VR interventions demonstrated high patient acceptability across studies. Where quantitative data were available, satisfaction scores were consistently high. For example, Van Doren et al.^38^ reported a mean satisfaction score of 8.55 out of 10, while Garland et al.^48^ achieved a Net Promoter Score of 71.43, indicating very high satisfaction. Greenwald et al.^46^ reported that 94% of patients enjoyed the experience. Qualitative feedback similarly emphasized positive experiences, with participants describing VR interventions as “calming and relaxing”^38^, “memorable” and “emotional”^42^, and “therapeutically promising”.^43^ Studies that used behavioral proxies for acceptability also showed strong engagement: one reported that 71% of eligible veterans chose to receive the VR headset^41^, and another maintained a near-100% completion rate.^40^

#### Tolerability.

Interventions were generally found to be safe and well-tolerated, with few adverse events associated with the virtual reality technology. While minor physiological side effects were reported, they rarely led to treatment discontinuation, with the exception of a few cases of motion sickness. The only reported adverse events were a panic attack that occurred 20 minutes after a session on drug cue exposure^48^ and a psychotic episode, though it was unclear if the event was linked to the VR intervention.^44^ Motion sickness induced by the VR environment (“simulator sickness” or “cybersickness”) was reported in three studies. Landhäußer et al.^47^ had three participants (5%) discontinue because of motion sickness. Garland et al.^48^ reported one instance of nausea (3%). Three of the five participants in Thaysen-Petersen et al.^49^ reported mild cybersickness. Six studies reported no instances of simulator sickness, while the remaining five did not report on simulator sickness.

### Implementation

#### Intended integration strategy.

All studies described VR experiences that were designed to be used as a complement to existing treatment, rather than used on a self-guided basis or as a treatment in themselves. However, ten of the experiences (71%) appeared to be described as primarily an “add-on” or adjunctive tool, meaning its intended use was *as an addition to* a standard treatment, and relatively distinct from it. The four remaining experiences seemed intended to be more embedded into treatment itself, meaning VR was used as a tool to help deliver a particular technique or help patients learn a skill that is often a typical part of those treatments but is done in a different way. For example, Thaysen-Petersen et al.^49^ immersed participants in restaurant environments using 360-degree videos in order to help participants identify unhelpful thoughts and beliefs related to these situations and practice coping skills as part of cognitive behavioral therapy for alcohol use disorder.

#### Barriers.

Many of the reviewed studies also identified several barriers to implementation that were unique to VR that emerged as they used it with patients. Common barriers included technical limitations, such as heavy headset weight and uncomfortable head-mounted display fits with some hardware systems.^37,38^ There were also concerns about sanitizing the headset between patients.^36^ Technical difficulties were also reported, including computational lag in complex environments like virtual supermarkets and gesture-tracking frustrations.^47^ Some studies that incorporated additional instruments, such as smartwatches or other physiological sensors, also reported technical problems that resulted in missing data.^39,48^ Other interventions required intensive involvement from clinical personnel, such as relying on a single therapist to manually animate avatars.^44^

## Discussion

In this study, we synthesized recently published research (2020–2025) describing VR experiences intended to improve SUD outcomes to explore their technical and clinical features and evaluate their readiness for implementation in community-based addiction treatment facilities. By considering the implementation readiness of these experiences, we hoped to assess whether more recent work was trending toward tools that were better-prepared for use in real-world clinical settings or whether they continue to rely on approaches that may be difficult to use outside of well-controlled research settings. Overall, we found that the majority of these recently-described experiences had substantial technical, procedural or conceptual limitations that could present significant barriers to their practical integration into routine clinical workflows. Although we recognize that there is value in testing more “experimental” approaches for VR applications that, if promising, can then be modified to ease implementation, we believe that doing so can be more difficult than designing with implementation in mind from the beginning.

Although we did not focus on evaluating the efficacy of VR as an approach to improving SUD outcomes or the methodological quality of included studies, our review echoes the findings of many previous reviews that there is very limited evidence about the benefit of VR-assisted treatments for SUD so far, largely because the rigor of the research in this area is poor.^16,17,23^ Few experiences have been tested beyond very small samples with extremely limited follow-ups or in studies that incorporated controls. Studies analyzed in this review had an average sample size of 40, the average follow-up was two months post-baseline, and only 43% incorporated any control. Given this, one of the most significant and pressing factors affecting VR’s readiness for implementation is likely the lack of evidence supporting its benefit. Past research suggests that this lack of evidence of VR’s impact on patient outcomes is among the top factors that would limit adoption among counselors and treatment facilities.^50^ Still, given that nearly all past reviews emphasize that research to date supports the *promise* of VR-assisted treatments for SUD^16,17,51^, evaluating other implementation-relevant characteristics is important to ensuring that these platforms are ready for clinical use if more robust evidence eventually supports their adoption.

### Platform characteristics

Characteristics of the VR experiences themselves and the hardware and software ecosystems they use are likely to affect their ease of use in clinical settings. For example, experiences that use VR ecosystems that require headsets to be tethered to a PC or hardware to be mounted in a specific space (e.g., base stations) may be particularly difficult to use in many treatment facilities due to the space constraints they create. Although tethered headsets have become increasingly rare in the consumer market, the studies included in this review suggest that research and clinical deployments may have been slower to adopt standalone alternatives: four studies (29%) appeared to use ecosystems with tethering constraints, although an equal number (n = 4) did not explicitly identify what VR system was used, requiring us to infer these characteristics from other language in publications (e.g., “attached to a gaming laptop” or “used in tethered mode”). Since tethered systems can only be used in a specific space, they can also create utilization bottlenecks, which could cap the value of adopting VR for facilities and create scheduling problems. Four experiences (29%) had even more complicated requirements, such as adjoining and private soundproof rooms, that could be very difficult for treatment facilities to accommodate.

Similarly, VR experiences that patients interact with by naturally moving within a boundary could present similar logistical challenges and could conflict with the preferences of this population. Having patients navigate experiences by physically moving around a space could add additional realism, but would come at the cost of requiring treatment facilities to maintain dedicated rooms of sufficient size to meet the experience’s specific spatial requirements. Past studies of counselor preferences suggest that having the space required to use VR is among their top concerns about adopting it.^50^ For SUD patients, navigating experiences through free movement could also decrease trust and hinder engagement, potentially because of the perceived safety risk of colliding with physical objects while essentially being “blindfolded” by the headset. Encouragingly, all but one experience was used in a stationary sitting position, which could suggest that more recent research is considering the realities of these contexts.

The recommended “dosage” of VR is also relevant to its “fit” with clinical settings, as well. The most common dose among reviewed studies was a single session ranging from six to 60 minutes, which is likely to be feasible in busy clinics. However, five experiences (36%) required patients to use VR for sessions of ≥ 60 minutes, which may be difficult to achieve in practice. Another five (36%) required patients to use VR for a total of 300–600 minutes over 8–24 sessions, protocols which could be especially difficult to execute in practice. Finally, the degree of interactivity involved in VR experiences could affect ease of implementation, given that more interactive environments require users to learn methods of interaction, such as hand gestures or controllers. This could increase patient orientation and training demands, although most patients orient to VR quickly, even without previous experience.^52^ Incorporating interactive elements could also boost the efficacy of these experiences, given evidence that experiences that include more interactivity are generally more immersive and are key to transitioning the user from an observer of the environment to having a sense of presence within it.^53–55^ Less interactive experiences like 360-degree video can effectively achieve place illusion, but are limited to passive, rotational observation, rather than providing reactive digital elements and agency that may be needed to perceive an environment as a real, responsive space. Of the studies we reviewed, nearly half were entirely passive, most often using exclusively 360-degree videos. This suggests that while interactive experiences are currently less common, the manageable increases in patient training they require could be a worthwhile trade-off for the heightened immersion and agency they provide.

### Therapeutic content

To maximize implementation readiness, SUD-focused VR experiences should augment or align with counseling approaches that (1) are evidence-based, (2) counselors and treatment facilities are familiar with, and (3) ideally, many counselors already regularly use. Without evidence of their efficacy or alignment with approaches that are already evidence-based, facilities and counselors may have little incentive to adopt them given the uncertain benefits they would provide patients. Past research shows that the strength of evidence supporting VR is one of the most important factors they consider when weighing whether or not to adopt it.^50^ VR experiences that counselors and facilities are unfamiliar with or do not regularly use may also introduce significant interest and training-related barriers among counselors. Of the 14 studies we reviewed, 43% were inspired at least partially by cue exposure therapy paradigms. Traditional cue exposure therapy paradigms, which involved gradually exposing patients to drug-related cues introduced during face-to-face interactions in the clinic, have received limited or inconsistent support in past research.^56^ Partly as a result, counselors and facilities may also be familiar with cue exposure techniques. It is possible, however, that VR-facilitated cue exposure could ultimately prove to be more effective than traditional cue exposure paradigms, particularly since VR-delivered cues can be presented in contexts where patients often encounter them (e.g., bars, social situations, home) rather than a clinic office. Nevertheless, additional research is needed to evaluate this possibility.

Roughly a third of VR experiences we reviewed were explicitly or directly inspired by well-known evidence-based treatment approaches^35^, namely CBT and mindfulness, augmenting techniques that are likely to be widely recognizable to many practicing addiction counselors. The remaining two-thirds involved techniques that were either likely to be entirely new to clinicians or a mixture of familiar and new concepts. While it may be feasible to train clinicians in the novel techniques these experiences support, and some protocols may require only a minimal training investment, the empirical evidence for a given experience’s efficacy would need to be compelling enough to warrant that effort. For facilities to invest the time and resources necessary to adopt a new approach, the potential improvement in patient outcomes would need to be clear and significant enough to outweigh the practical costs of implementation.

Another potential application involves using VR as a facilitator of more established interventions. For example, interest is growing in psychedelic-assisted treatments for SUD and related psychiatric conditions.^57^ Since past research has shown that VR can induce altered states of consciousness that may be therapeutically relevant, such as ego attenuation and self-transcendence^55^, and cause effects characteristic of psychedelics,^56^ these characteristics have led some to explore its potential as an adjunct to, and potentially as a non-pharmacological alternative for evoking, therapeutically relevant altered states.^58^ Although this literature falls outside the scope of the current review, it suggests that future VR interventions for SUD need not be limited to augmenting existing behavioral approaches and may eventually offer entirely novel mechanisms of therapeutic change.

### Clinician roles and training

Another factor that affects the degree of counselor training required to implement the VR experiences we reviewed is the extent to which they depend on clinicians’ active involvement with patients while they are engaged with it or afterward (e.g., offering coaching while patients navigate the experience or helping them process it afterward). Only four of the 14 studies we reviewed involved such an active role for clinicians (29%), with the remainder being primarily self-guided. Of those that required active roles of counselors, only one^49^ was specific about the time commitment needed for training, which was an extensive 49 hours. However, this study involved training nurses to use VR as part of a larger manualized CBT treatment, and so, much of that training may have been focused on non-VR techniques and could be more efficient among counselors who are already familiar with CBT techniques. Although it is not clear how much training was involved for the other studies that required active clinician involvement, many described complex and novel skills that would seem to require significant time commitment. More self-guided experiences generally seemed to have much lower training requirements, but still often involved learning to provide VR experiences in specific sequences, to orient patients to VR, or respond to issues that arose.

Importantly, while experiences that require more active clinician involvement inevitably increase training requirements and thus barriers to implementation, there may be important benefits to actively involving counselors that ultimately aid implementation and sustainability rather than hinder it. For instance, counselors who actively coach patients through an experience may feel a greater sense of professional investment in VR and buy-into it more. Active involvement also allows clinicians to help patients process and contextualize what they experienced, which could help consolidate VR’s effects well after the experience is over. This also may reduce the risk that VR becomes a siloed or passive activity that feels disconnected from the rest of treatment. Entirely self-directed experiences may be easier to adopt at first but could suffer from poorer long-term engagement if they are not reinforced by a provider. Ultimately, it may be best to design these tools to keep technical training requirements as low as possible while still ensuring there is a clear, functional role for the counselor to play in the therapeutic process.

### Patient training, acceptability, and usability

Experiences that have more complex patient training requirements may be more difficult to implement in practice, as well. However, half of the studies we reviewed required relatively minimal patient training in order to use the VR systems, often involving a brief orientation to its controls and to the purpose of the intervention. The remaining half involved more significant patient training needs. In addition to increasing the burden involved for clinicians, experiences that require more patient orientation also pose greater risk of disrupting typical session workflows, which could lead to discontinuation. While past studies have shown this concern is not high on the list for counselors^50^, it could still be an important factor that arises as they gain real experience using VR in practice.

Findings of the reviewed studies also largely echoed the growing literature showing that SUD patients find VR acceptable and give it high satisfaction ratings across a variety of experiences^57,58^. Although infrequently reported, tolerability was also generally good, with mostly minor side effects related to simulator sickness affecting 3–5% of participants, consistent with past research showing that these incidents are low.^59^ It is important to note that the frequency and severity of simulator sickness is also associated with the VR ecosystems researchers use, with more modern systems having generally better ergonomic profiles than other systems.^60–62^ As such, it may be that future interventions that adopt more modern systems will report even lower rates of simulator sickness. High patient interest and minimal tolerability issues are critical implementation facilitators that could be used to address clinicians’ reservations and encourage facilities to move toward adoption. By highlighting that patients are enthusiastic about its use, advocates could frame VR as a tool that may increase patient engagement in treatment.

### Other implementation barriers

Several of the reviewed studies also identified other implementation barriers that are unique to VR, but are largely addressable. For example, two identified headset ergonomics (weight and fit) as issues reported by patients, but both used less common VR hardware systems that may be heavier and could be harder to fit appropriately. Likewise, past real-world pilots have identified several implementation strategies to address issues like equipment sanitizing, hygiene, and equipment management by using point-of-care VR carts that are regularly cleaned and maintained.^63^ Finally, our review found that, while paired devices for biomonitoring (e.g., heart rate, skin conductance) were common, their added value was often unclear and may have introduced significant technical hurdles and logistical complexity. Beyond these practical barriers, cost and equity considerations remain largely unaddressed in the reviewed literature. Though several papers noted the potential for self-guided, low-cost VR to expand access in under-resourced settings, none studied these platforms in underserved populations. Cost, technical support, and unclear reimbursement pathways remain meaningful barriers that may place VR out of reach for these settings and populations.

### Design implications

Given the above findings, we offer the following suggestions for researchers and developers designing VR interventions for SUD treatment that could increase implementation readiness:

1. Design for portable, standalone hardware that minimizes additional infrastructure requirements.
2. Keep session length, total treatment dose, and training burden realistic for community SUD treatment settings, where long or complex protocols are unlikely to be sustained.
3. Ground therapeutic content in evidence-based practices and integrate VR as a clear component of the therapeutic process rather than a standalone activity.
4. Favor interactive designs over passive approaches, as the modest increase in patient orientation needs may be outweighed by higher immersion and interest.

### Limitations

Several limitations of this review are important to note. First, because this was a rapid review, we only synthesized studies published within the last five years. While it is possible that older research may have described platforms with higher implementation readiness, older hardware systems typically present more technical and logistical barriers, so we do not believe this is likely to have substantially affected our conclusions. Second, we focused exclusively on peer-reviewed publications and did not include “grey literature,” such as technical reports, white papers, or product manuals, and so may have missed emerging implementation strategies or hardware configurations (e.g., from the private sector). Third, we focused primarily on characteristics of the VR platform itself (the “innovation domain”^26^) because these details were most consistently reported in the literature, as well as some characteristics of typical addiction treatment settings (the “inner setting”). As a result, this review did not fully capture other critical factors that play an important role in implementation, such as organizational climate, leadership support, or reimbursement pathways. Fourth, several studies provided incomplete details regarding hardware, software, content, and clinical goals, requiring us to occasionally infer these characteristics from other descriptive language in the publications, which may have led to some inaccuracies. In particular, hardware specifications were unspecified in four studies, which limited our ability to draw conclusions about tethering requirements and infrastructure needs for those platforms. However, we do not believe this meaningfully changed our overall conclusions, as the general pattern of findings was consistent across studies regardless of whether hardware was explicitly reported. Fifth, we chose not to conduct a formal quality assessment, as standard tools evaluate efficacy-related rigor, which was not the focus of this review. However, readers should note that the quality of the underlying evidence base is limited, and conclusions about implementation readiness are based on how platforms were described rather than implementation outcomes data. Many included studies involved small samples, highly specific populations, or inpatient treatment settings, which limits the transferability of findings to other contexts.

## Conclusion

In summary, the most important next step for VR interventions for SUD is to develop a clear and compelling evidence base for the clinical benefits of VR-assisted treatments for SUD. Should this research demonstrate clear therapeutic value, their adoption into routine clinical practice would likely depend on how well these experiences align with the practical needs of addiction treatment facilities. Our review found that while many recently-developed VR experiences for SUD are safe and acceptable to patients, many systems relied on immobile hardware and required additional equipment or infrastructure that are likely to create logistical barriers in community treatment settings. Furthermore, many existing platforms were not explicitly rooted in common evidence-based practices, which could create barriers by requiring counselors to learn unfamiliar methods rather than leveraging the skills they are already familiar with. Similarly, based on the descriptive characteristics in the reviewed studies, most reviewed platforms appeared to function as supplemental “add-ons” rather than integrated clinical tools, an approach that could risk the technology becoming a dispensable accessory rather than a staple of evidence-based care. Ultimately, for VR to move beyond research, developers should design experiences with the implementation context in mind to ensure they align with the practical realities of the settings in which they will be used.

## Supplementary Material

Supplementary Files

This is a list of supplementary files associated with this preprint. Click to download.

- PRISMARR2020checklistcompleted.docx

## Figures and Tables

**Table 1.** Mapping of coded platform and implementation characteristics to CFIR constructs.

Coded characteristic	CFIR Domain	CFIR Construct
**Platform & system characteristics**		
Hardware ecosystem (standalone vs. PC-tethered)*	Innovation	Design
	Inner Setting	IT Infrastructure
Additional infrastructure (soundproof rooms, free-walking space)	Innovation	Complexity
	Inner Setting	Physical Infrastructure
Recommended dosage (session length & number)*	Innovation	Complexity
	Inner Setting	Compatibility
In-session navigation (stationary vs. free physical movement)*	Innovation	Complexity
	Inner Setting	Physical Infrastructure
Interactivity (active vs. passive)*	Innovation	Complexity
		Design
Common content elements	*Used for descriptive purposes, not mapped to CFIR*	
**Therapeutic content**		
Underlying theoretical model	*Used for descriptive purposes, not mapped to CFIR*	
Alignment with evidence-based practices	Innovation	Evidence-Base
**Clinician roles & training**		
Therapist guidance during experience*	Inner Setting	Compatibility
	Individuals	Capability: Deliverers
Therapist training burden	Innovation	Complexity
	Inner Setting	Capability: Deliverers
**Patient training, acceptability & usability**		
Patient training needs	Innovation	Complexity
	Individuals	Capability: Recipients
Patient acceptability & usability*	Individuals	Recipients: Motivation
	Innovation	Design
Tolerability	*Used for descriptive purposes, not mapped to CFIR*	
**Implementation context & other barriers**		
Intended workflow fit (adjunct vs. embedded)	Inner Setting	Compatibility
VR-specific barriers (headset ergonomics, paired biomonitoring, etc.)	Innovation	Complexity
		Design

Asterisk (*) indicates variables that represent VR-specific operationalizations of CFIR constructs as adapted by the authors.

**Table 2.** Summary of study characteristics.

Source	Study design	Setting	Substance of focus	Sample size (N)	Key demographics	Follow-up period	Outcome measures
Faraj et al. (2021)	Pilot (Single-arm)	Outpatient (MAT)	OUD	15	73% female; majority >50 yrs; 53% white	None	Pain (fMRI pain neuromatrix activation/connectivity) Cortisol and C-reactive protein (CRP) (Saliva biomarkers) Self-reported pain and affective symptoms (Visual Analog Scales)
Garland et al. (2024)	Phase 1 Pilot (Single-arm)	Outpatient (MAT)	OUD	28	78% male; mean age 38.5 yrs; 93% white	None	Feasibility & Engagement (enrollment/retention rates) Acceptability (likelihood to recommend/Net Promoter Score) Usability (NRS scale)
Ghiţă et al. (2025)	RCT (2-arm)	Outpatient	AUD	85	60% male; mean age 52 yrs	1 year	Multidimensional Alcohol Craving Scale (MACS-VR) State-Trait Anxiety Inventory (STAI-State)
Giguère et al. (2024)	Pilot Clinical Trial (Single-arm)	Outpatient & Inpatient	Cannabis + severe mental disorders	32	84% male; mean age 37 yrs; 88% white	1 year	Cannabis ingestion quantity & frequency (Timeline Follow-Back and urinary THC-COOH quantification)
Greenwald et al. (2024)	Pilot (Single-arm)	Inpatient (acute stabilization)	OUD	32	72% male; mean age 40 yrs; 59% white	None	Treatment completion rate (administrative records) Days on the unit (administrative records) Suitability Evaluation Questionnaire (SEQ-Modified)
Hargett et al. (2022)	Pilot (Single-arm)	Inpatient (orthopedic/trauma)	OUD or tolerance	11	55% female; mean age 53.5 yrs; 73% white	None	Numeric Pain Scale
Huang et al. (2025)	RCT (3-arm)	Inpatient (compulsory)	Methamphetamine	89	100% male; mean age 36 yrs	None	Tonic craving, Cue-induced craving (Visual Analog Scale)
Ji et al. (2023)	RCT (2-arm)	Inpatient (compulsory)	Methamphetamine	60	100% female; mean age 23 yrs	1 month	Subjective craving (Visual Analog Scale) Physiological responses (heart rate, blood pressure)
Landhäußer et al. (2025)	Pilot RCT (6-arm factorial)	Inpatient & day-clinic	AUD	58	None reported	None	Negative affect (PANAS-NA) State anxiety (STAIS-5) Alcohol craving (AUQ) Stress appraisal (PASA) Sense of presence (igroup Presence Questionnaire (IPQ)) Physiological responses (heart rate, electrodermal activity)
Shen et al. (2022)	Pilot (Single-arm)	Outpatient & recovery housing	Mixed SUD	21	71% male; mean age 34 yrs; 90% white	30 days	Future self-continuity (Similarity and Connectedness Euler scales) Delay discounting (Adjusting-amount DD task)
Thaysen-Petersen et al. (2024)	Pilot RCT (2-arm)	Outpatient	AUD	9	56% male; median age 46 yrs	1 month	Feasibility (Drop-out rate, psychological reactions, and Simulator Sickness Questionnaire (SSQ))
Van Doren et al. (2024)	Pilot (Single-arm)	Inpatient (VA)	Mixed SUD	20	85% male; mean age 51 yrs; 41% white	None	Acceptability, usability & satisfaction State emotion (Visual Analog Scale) Mindfulness (Toronto Mindfulness Scale, adapted) Sense of presence
Wiese et al. (2024)	Program evaluation	Inpatient (VA)	Mixed SUD	51	94% male; mean age 56 yrs; 67% Black	Inpatient treatment completion	Successful completion of treatment (administrative records)
Zhang et al. (2023)	RCT (2-arm)	Inpatient	AUD	44	100% male; mean age 35 yrs; 100% Han nationality	None	Subjective craving (Visual Analog Scale) Physiological responses (heart rate, skin conductance, and respiration)

**Table 3.** Summary reference of the platform characteristics of each described VR experience.

Source	Hardware	Navigation	Interactivity level	Infrastructure requirements	Peripherals	Content elements	Session dosage
Faraj et al. (2021)	Windows Mixed Reality	Stationary (active upper body)	Active	Tethered to PC	-	Internal “beast,” child avatars, and nature scenes	16–24 sessions × 30 min
Garland et al. (2024)	Unspecified	Stationary (with controllers)	Active	Headset only	Photoplethysmography-enabled smartwatch	Nature scenes, substance cue exposure	8 sessions × 45–60 min
Ghiţă et al. (2025)	Oculus Rift S	Stationary (with controllers)	Active	Tethered to PC	Scent cue (alcohol exposure)	High-risk social scenes with substance cue exposure (e.g. bar, restaurant)	6 sessions × ~60 minutes
Giguère et al. (2024)	Unspecified	Stationary	Active	Specialized space, Tethering needs unspecified	Noise-cancelling headphones	High-risk social scenes; therapist-animated avatars	≥8 sessions × 60–90 min
Greenwald et al. (2024)	Meta Quest 2	Stationary (with controllers)	Active	Headset only	-	Interactive 3D art, problem-solving games, and social dialogue	1–2 sessions × ~20 min
Hargett et al. (2022)	Oculus Go	Stationary	Passive	Headset only	-	Nature scenes	1 session × 10 min
Huang et al. (2025)	Clinical-specific (from Hangzhou Xinjing Technology Co. Ltd)	Stationary	Passive	Central controller computer, Tethering needs unspecified	-	Substance cue exposure, high-risk social scenes, aversive consequences, nature scenes	16 sessions × 22 min
Ji et al. (2023)	PICO G2	Stationary	Passive	Headset only	Blood pressure monitor	Substance cue exposure, nature scenes	8 sessions × 60 min
Landhäußer et al. (2025)	Meta Quest 3	Physical free movement	Active	Tethered to PC, Specialized space	Empatica E4 wristband	High-risk social scenes, nature scenes, individualized substance cue exposure	1 session × ~60 min
Shen et al. (2022)	Samsung Odyssey	Stationary (standing/seated)	Semi-active (limited decision points)	Tethered to PC, Specialized space	Park bench, scent diffuser (new mown grass)	Personalized age-progressed future self avatars	1 session × ~10 min
Thaysen-Petersen et al. (2024)	Unspecified	Stationary	Active	Tethering needs unspecified	-	High-risk social scenes	3 sessions × 45–60 min
Van Doren et al. (2024)	Clinical-specific (REAL systems i-series)	Stationary	Passive	Headset only	-	Nature scenes	1 session × ~60 minutes
Wiese et al. (2024)	Unspecified	Virtual movement	Passive	Tethering needs unspecified	-	Nature scenes, psychoeducational content	Self-paced
Zhang et al. (2023)	Oculus Quest	Stationary	Passive	Headset only	Multiparameter biofeedback system (Infiniti3000A), Scent cue (alcohol exposure)	High-risk social scenes	8 sessions × 8 min

**Table 4.** Therapeutic elements of the 14 interventions in this review.

Intervention	Cue exposure & extinction	Mindfulness Cognitive-behavioral	Other	*Aligned with existing evidence-based practices* ^1^
Faraj et al. (2021)		X	X	Some
Garland et al. (2024)	X	X		Yes
Ghiţă et al. (2025)	X			No
Giguère et al. (2024)				Yes
Greenwald et al. (2024)		X		Some
Hargett et al. (2022)		X		Yes
Huang et al. (2025)	X			No
Ji et al. (2023)	X	X		No
Landhäußer et al. (2025)	X			No
Shen et al. (2022)			X	No
Thaysen-Petersen et al. (2024)		X		Yes
Van Doren et al. (2024)		X		Yes
Wiese et al. (2024)		X		Some
Zhang et al. (2023)	X			No

Note.

1 Interventions were coded as “aligned” with evidence-based practices if they explicitly and directly mentioned drawing inspiration from an approach to treatment that was mentioned in the National Institute on Drug Abuse’s *Principles of Addiction Treatment*^34^ or McGovern and Carroll’s^35^ review of evidence-based practices for substance use disorders.

## Data Availability

This rapid review synthesized data from previously published studies. All included studies are cited in the reference list. No new datasets were generated or analyzed during the current study. A full list of included articles and coding decisions is available from the corresponding author upon request.
